# Supplementary material for: Modulation of GluA2–γ5 synaptic complex desensitization, polyamine block and antiepileptic perampanel inhibition by auxiliary subunit cornichon-2
Source: Nat Struct Mol Biol. 2023 Aug 31;30(10):1481–94. doi: 10.1038/s41594-023-01080-x (PMC10584687; doi:10.1038/s41594-023-01080-x)
Supplement: Supplementary file 1 — Supplementary Figs. 1–5. [file 41594_2023_1080_MOESM1_ESM.pdf]

nature structural & molecular biology

Article

https://doi.org/10.1038/s41594-023-01080-x

Modulation of GluA2–γ5 synaptic complex desensitization, polyamine block and antiepileptic perampanel inhibition by auxiliary subunit cornichon-2

In the format provided by the authors and unedited

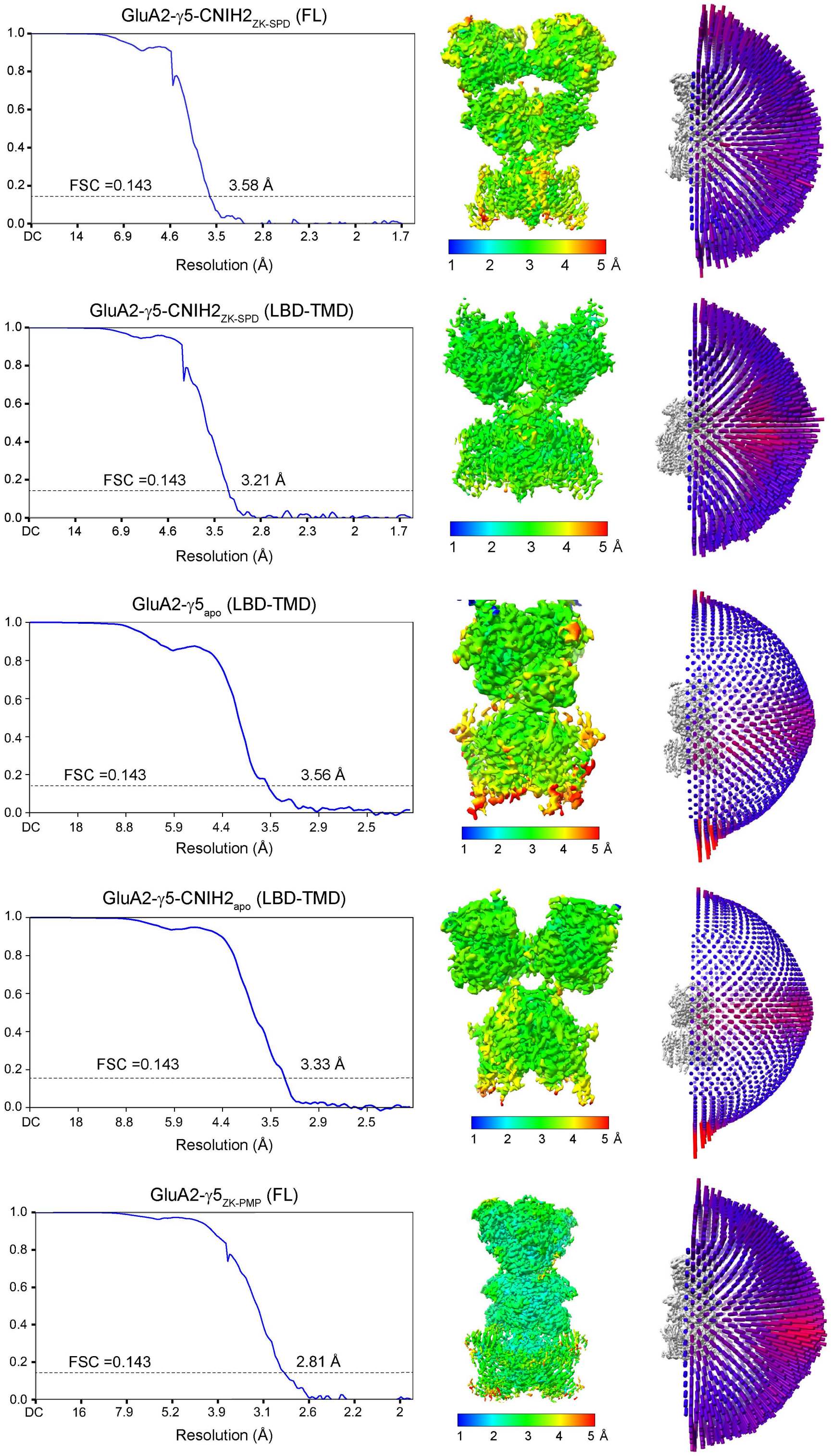

Supplementary Figure 1 | Characteristics of cryo-EM reconstructions in the presence of ZK200775, in the apo condition and in the presence of perampanel. Shown are FSC curves for full-length (FL) complexes and their LBD-TMD portion calculated between half maps, with the resolution range estimated using the FSC = 0.143 criterion (left), local resolution presented as coloring of the cryo-EM maps in Chimera (middle) and Euler angle distribution of particles contributing to the final reconstruction with larger red cylinders representing orientations comprising more particles (right).

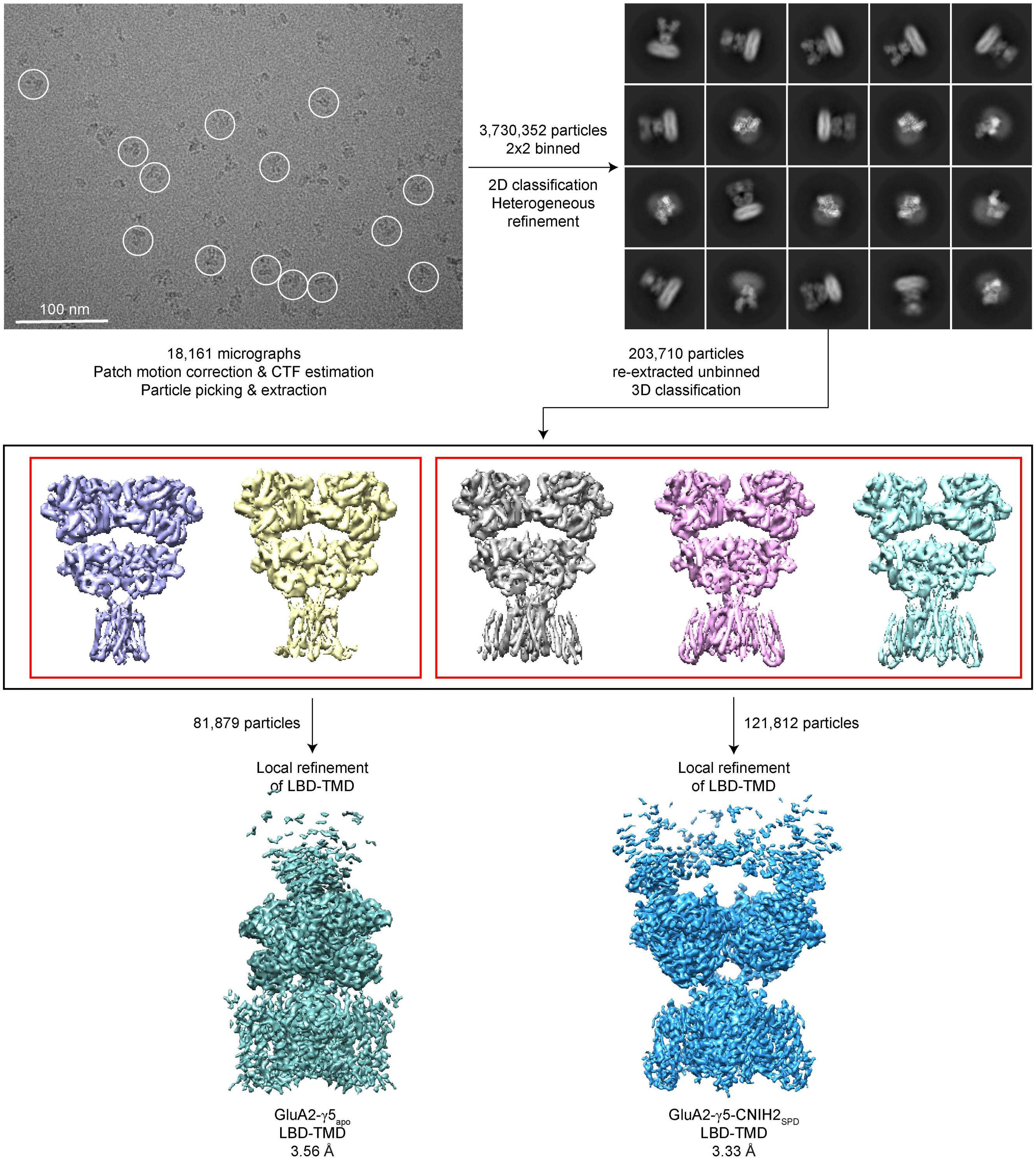

Supplementary Figure 2 | Cryo-EM data processing workflow for GluA2-γ5 and GluA2-γ5-CNIH2 in the apo state. A representative micrograph for the protein subjected to cryo-EM in the absence of ligands (apo condition) shows example particles circled in white.

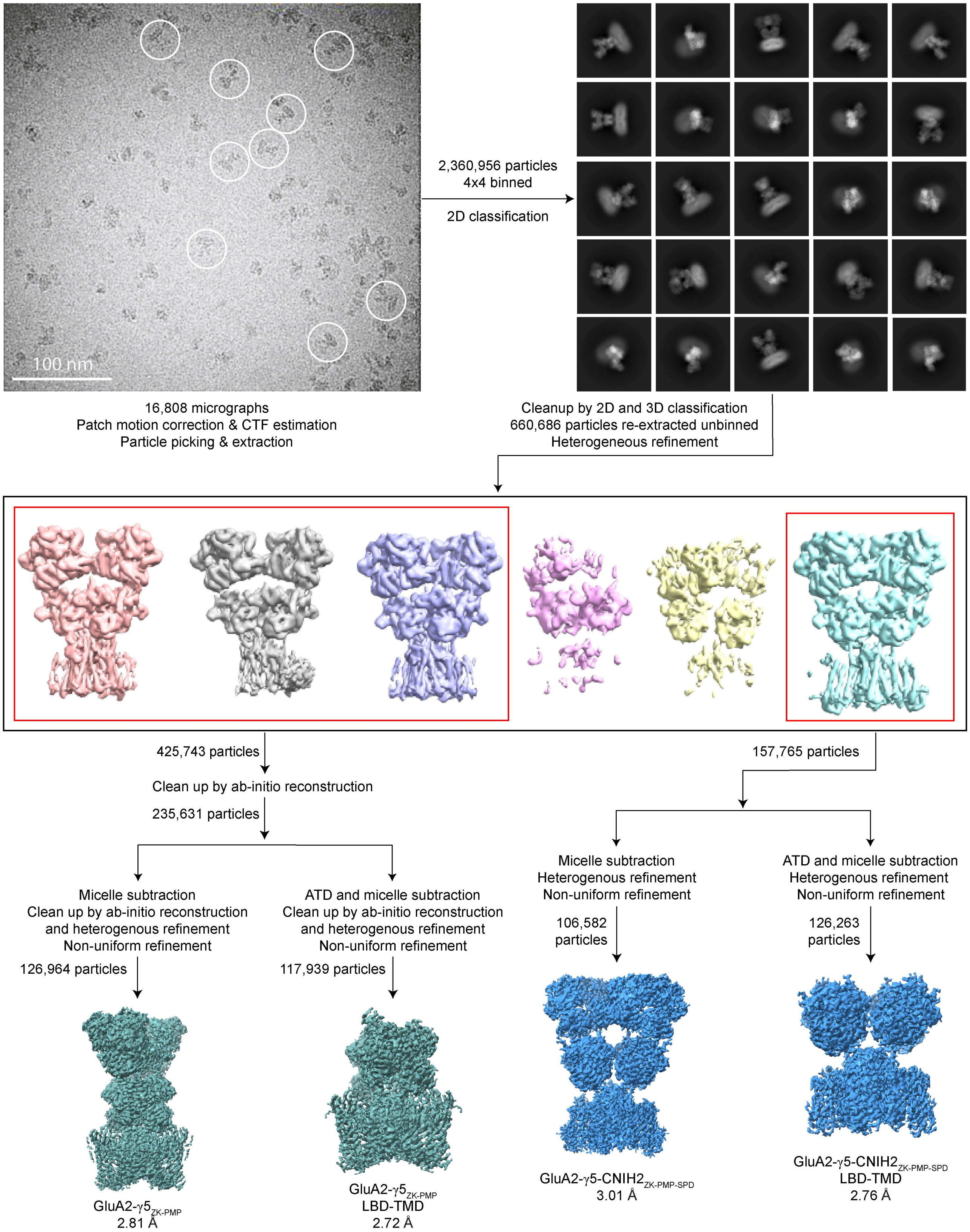

Supplementary Figure 3 | Cryo-EM data processing workflow for GluA2-γ5 and GluA2-γ5-CNIH2 in the presence of the antiepileptic drug perampanel. A representative micrograph for the protein subjected to cryo-EM in the presence of 100 μM ZK and 100 μM PMP shows example particles circled in white.

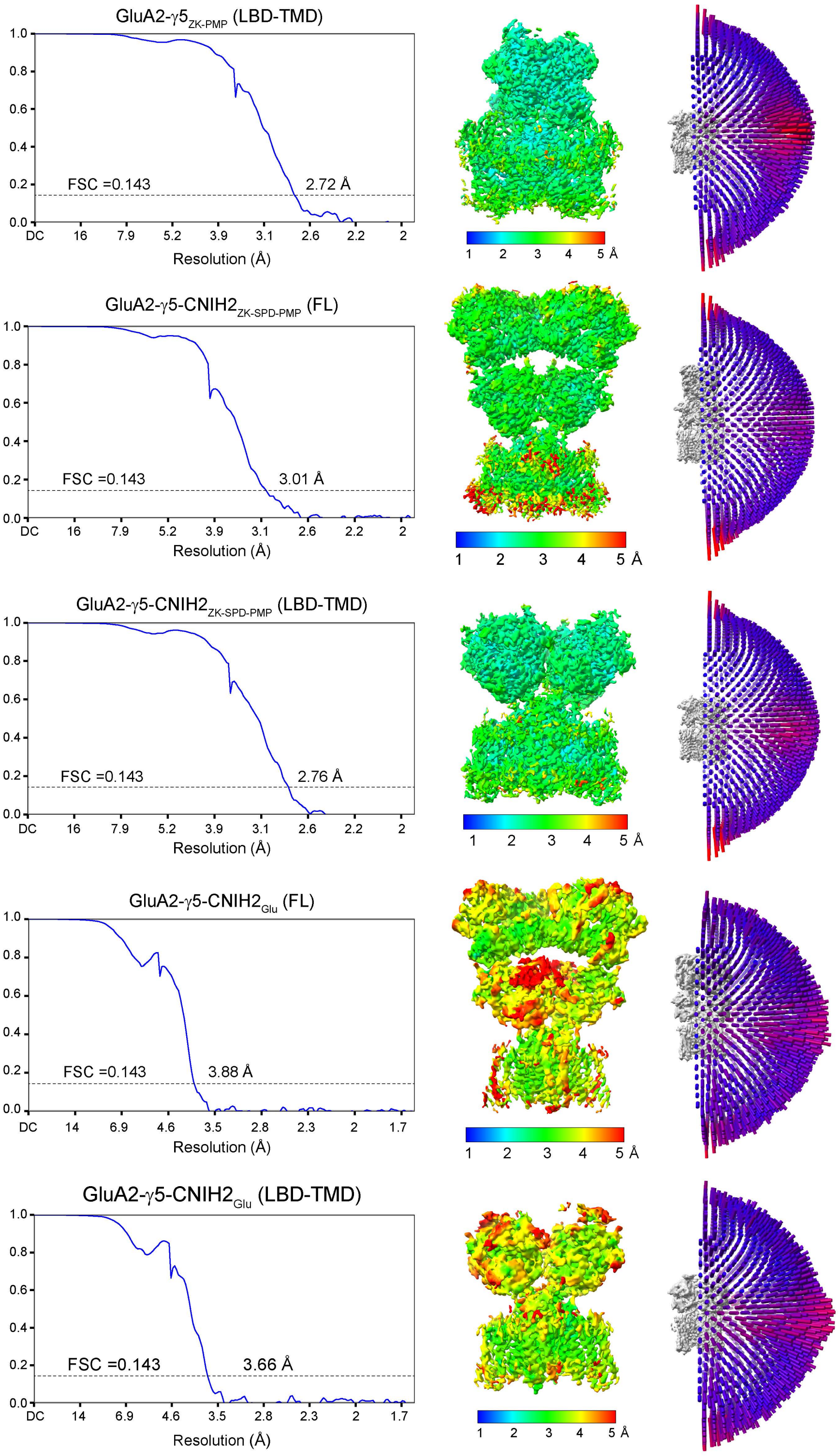

Supplementary Figure 4 | Characteristics of cryo-EM reconstructions in the presence of perampanel and glutamate. Shown are FSC curves for full-length (FL) complexes and their LBD-TMD portion calculated between half maps, with the resolution range estimated using the FSC = 0.143 criterion (left), local resolution presented as coloring of the cryo-EM maps in Chimera (middle) and Euler angle distribution of particles contributing to the final reconstruction with larger red cylinders representing orientations comprising more particles (right).

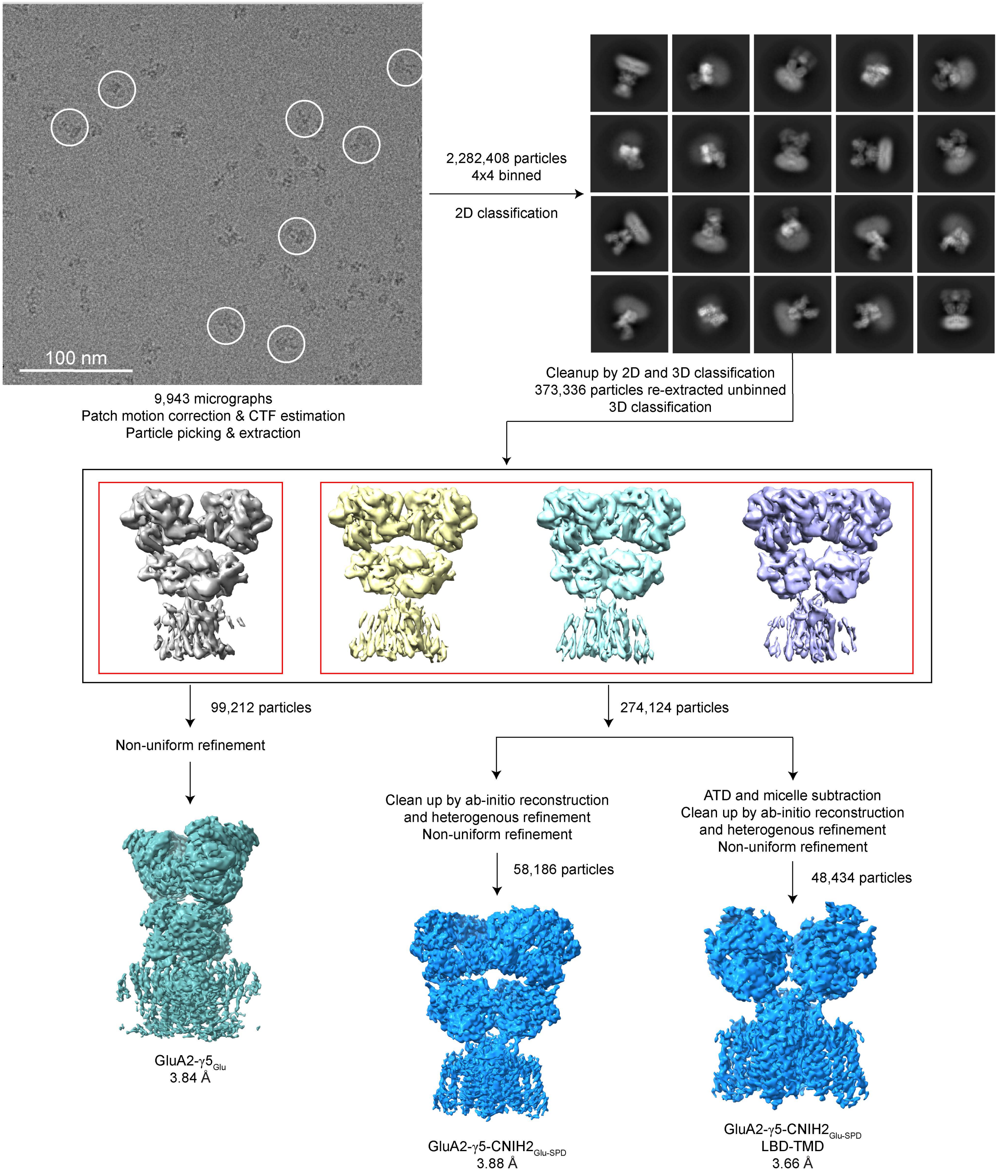

Supplementary Figure 5 | Cryo-EM data processing workflow for GluA2-γ5 and GluA2-γ5-CNIH2 in the presence of glutamate. A representative micrograph for the protein subjected to cryo-EM in the presence of 10 mM Glu shows example particles circled in white.
