# Supplementary material for: Modulation of GluA2–γ5 synaptic complex desensitization, polyamine block and antiepileptic perampanel inhibition by auxiliary subunit cornichon-2
Source: Nat Struct Mol Biol. 2023 Aug 31;30(10):1481–94. doi: 10.1038/s41594-023-01080-x (PMC10584687; doi:10.1038/s41594-023-01080-x)
Supplement: Supplementary file 2 — Reporting Summary [file 41594_2023_1080_MOESM2_ESM.pdf]

nature portfolio

Corresponding author(s): Alexander Sobolevsky

Last updated by author(s): Jul 14, 2023

Reporting Summary

Nature Portfolio wishes to improve the reproducibility of the work that we publish. This form provides structure for consistency and transparency in reporting. For further information on Nature Portfolio policies, see our Editorial Policies and the Editorial Policy Checklist.

Statistics

For all statistical analyses, confirm that the following items are present in the figure legend, table legend, main text, or Methods section.

n/a	Confirmed	
☐	☒	The exact sample size (*n*) for each experimental group/condition, given as a discrete number and unit of measurement
☐	☒	A statement on whether measurements were taken from distinct samples or whether the same sample was measured repeatedly
☐	☒	The statistical test(s) used AND whether they are one- or two-sided<br>*Only common tests should be described solely by name; describe more complex techniques in the Methods section.*
☒	☐	A description of all covariates tested
☒	☐	A description of any assumptions or corrections, such as tests of normality and adjustment for multiple comparisons
☐	☒	A full description of the statistical parameters including central tendency (e.g. means) or other basic estimates (e.g. regression coefficient) AND variation (e.g. standard deviation) or associated estimates of uncertainty (e.g. confidence intervals)
☐	☒	For null hypothesis testing, the test statistic (e.g. *F*, *t*, *r*) with confidence intervals, effect sizes, degrees of freedom and *P* value noted<br>*Give P values as exact values whenever suitable.*
☒	☐	For Bayesian analysis, information on the choice of priors and Markov chain Monte Carlo settings
☒	☐	For hierarchical and complex designs, identification of the appropriate level for tests and full reporting of outcomes
☒	☐	Estimates of effect sizes (e.g. Cohen's *d*, Pearson's *r*), indicating how they were calculated

Our web collection on statistics for biologists contains articles on many of the points above.

Software and code

Policy information about availability of computer code

Data collection	Leginon 3.5, SerialEM 4.0, EPU 2
Data analysis	RELION 4.0, MotionCor2, CTFFIND4.1, cryoSPARC 4.2.0, cryoSPARC 4.2.1, UCSF Chimera 1.16, UCSF ChimeraX 1.3, COOT 0.9.8.1, PHENIX 1.18, pyMOL 2.5.2, HOLE 2.1, Origin 9.1.0, , Python SciPy 1.5.4, GraphPad Prism 9.4.1, Thermo Qual BrowserTM 4.0.27.19, pCLAMP 10.2, Clampfit10.3, DynDom 1.5

For manuscripts utilizing custom algorithms or software that are central to the research but not yet described in published literature, software must be made available to editors and reviewers. We strongly encourage code deposition in a community repository (e.g. GitHub). See the Nature Portfolio guidelines for submitting code & software for further information.

Data

Policy information about availability of data

All manuscripts must include a data availability statement. This statement should provide the following information, where applicable:

- Accession codes, unique identifiers, or web links for publicly available datasets
- A description of any restrictions on data availability
- For clinical datasets or third party data, please ensure that the statement adheres to our policy

The cryo-EM density maps have been deposited to the Electron Microscopy Data Bank (EMDB) under the accession codes EMD-40741 [https://www.ebi.ac.uk/emdb/EMD-40741] (full-length GluA2-γ5-CNIH2ZK-SPD), EMD-40742 [https://www.ebi.ac.uk/emdb/EMD-40742] (LBD-TMD of GluA2-γ5-CNIH2ZK-SPD), EMD-40743

[https://www.ebi.ac.uk/emdb/EMD-40743] (LBD-TMD for GluA2-γ5-CNIH2SPD), EMD-40744 [https://www.ebi.ac.uk/emdb/EMD-40744] (LBD-TMD for GluA2-γ5apo), EMD-40745 [https://www.ebi.ac.uk/emdb/EMD-40745] (full-length GluA2-γ5-CNIH2ZK-PMP-SPD), EMD-40746 [https://www.ebi.ac.uk/emdb/EMD-40746] (LBD-TMD for GluA2-γ5-CNIH2ZK-PMP-SPD), EMD-40747 [https://www.ebi.ac.uk/emdb/EMD-40747] (full-length GluA2-γ5ZK-PMP), EMD-40748 [https://www.ebi.ac.uk/emdb/EMD-40748] (LBD-TMD for GluA2-γ5ZK-PMP), EMD-40749 [https://www.ebi.ac.uk/emdb/EMD-40749] (full-length GluA2-γ5-CNIH2Glu-SPD), and EMD-40750 [https://www.ebi.ac.uk/emdb/EMD-40750] (LBD-TMD for GluA2-γ5-CNIH2Glu-SPD). The atomic coordinates have been deposited to the Protein Data Bank (PDB) under the accession codes 8SS2 [https://doi.org/10.2210/pdb8SS2/pdb] (full-length GluA2-γ5-CNIH2ZK-SPD), 8SS3 [https://doi.org/10.2210/pdb8SS3/pdb] (LBD-TMD of GluA2-γ5-CNIH2ZK-SPD), 8SS4 [https://doi.org/10.2210/pdb8SS4/pdb] (LBD-TMD for GluA2-γ5-CNIH2SPD), 8SS5 [https://doi.org/10.2210/pdb8SS5/pdb] (LBD-TMD for GluA2-γ5apo), 8SS6 [https://doi.org/10.2210/pdb8SS6/pdb] (full-length GluA2-γ5-CNIH2ZK-PMP-SPD), 8SS7 [https://doi.org/10.2210/pdb8SS7/pdb] (LBD-TMD for GluA2-γ5-CNIH2ZK-PMP-SPD), 8SS8 [https://doi.org/10.2210/pdb8SS8/pdb] (full-length GluA2-γ5ZK-PMP), 8SS9 [https://doi.org/10.2210/pdb8SS9/pdb] (LBD-TMD for GluA2-γ5ZK-PMP), 8SSA [https://doi.org/10.2210/pdb8SSA/pdb] (full-length GluA2-γ5-CNIH2Glu-SPD), and 8SSB [https://doi.org/10.2210/pdb8SSB/pdb] (LBD-TMD for GluA2-γ5-CNIH2Glu-SPD). The atomic coordinates under the accession codes 6DM1 [https://doi.org/10.2210/pdb6DM1/pdb], 7OCE [https://doi.org/10.2210/pdb7OCE/pdb], 5WEO [https://doi.org/10.2210/pdb5WEO/pdb], and 7R5Z [https://doi.org/10.2210/pdb7R5Z/pdb] were used for model building and structural comparisons. Source data are provided with this paper.

Human research participants

Policy information about studies involving human research participants and Sex and Gender in Research.

Reporting on sex and gender	N/A
Population characteristics	N/A
Recruitment	N/A
Ethics oversight	N/A

Note that full information on the approval of the study protocol must also be provided in the manuscript.

Field-specific reporting

Please select the one below that is the best fit for your research. If you are not sure, read the appropriate sections before making your selection.

☒ Life sciences ☐ Behavioural & social sciences ☐ Ecological, evolutionary & environmental sciences

For a reference copy of the document with all sections, see nature.com/documents/nr-reporting-summary-flat.pdf

Life sciences study design

All studies must disclose on these points even when the disclosure is negative.

Sample size	Amount of cryo-EM data collected was limited by time allocation at the microscopes. For electrophysiological experiments, we performed all measurements five times or more. The reported sample size is based on published research by us and others, and is sufficient to obtains reproducible and reliable data in HEK cells using AMPA receptors.
Data exclusions	No data has been excluded.
Replication	No replication attempts have failed. Cryo-EM data collections were performed during continuous two-day data collection sessions and were consistent from the beginning to the end. A replication of the cryo-EM data collection was therefore not necessary or economically justifiable. In electrophysiological experiments, we made at least five independent replicates for each construct.
Randomization	Samples were not randomized; it is not technically or practically feasible to do so for cryo-EM or patch-clamp studies. Covariant control is not economically viable in cryo-EM data collections. Covariant control was alos not possible in electrophysiological experiments due to the need to transfect with predetermind cDNAs and optimize protein expression for individual constructs.
Blinding	Researchers were not blinded; it is not technically or practically feasible to do so for cryo-EM or electrophysiological experiments. It is not economically viable to blind cryo-EM collections. For electrophysiological experiments, researchers conducting the studies were also in charge of cell as well as protein expression optimization for individual constructs in order to achieve recordings or transfected cells in these studies. These circumstances made blinding not possible.

Reporting for specific materials, systems and methods

We require information from authors about some types of materials, experimental systems and methods used in many studies. Here, indicate whether each material, system or method listed is relevant to your study. If you are not sure if a list item applies to your research, read the appropriate section before selecting a response.

Materials & experimental systems

n/a	Involved in the study
☒	☐ Antibodies
☐	☒ Eukaryotic cell lines
☒	☐ Palaeontology and archaeology
☒	☐ Animals and other organisms
☒	☐ Clinical data
☒	☐ Dual use research of concern

Methods

n/a	Involved in the study
☒	☐ ChIP-seq
☒	☐ Flow cytometry
☒	☐ MRI-based neuroimaging

Eukaryotic cell lines

Policy information about cell lines and Sex and Gender in Research

Cell line source(s)	HEK293S GnTI-, ATCC, Cat#CRL-3022<br>Sf9, Gibco, Cat#12659017<br>HEK 293, ATCC, Cat#CRL-1573
Authentication	None of the cell lines used have been authenticated.
Mycoplasma contamination	The cell lines used have been tested for mycoplasma contamination by the providers (negative results) but have not been retested in the lab.
Commonly misidentified lines<br>(See ICLAC register)	No commonly misidentified lines were used in this study.
